# Phenotypic Detection of Metallo-**β**-Lactamase in Imipenem-Resistant *Pseudomonas aeruginosa*

**DOI:** 10.1100/2012/654939

**Published:** 2012-06-18

**Authors:** Yalda Khosravi, Mun Fai Loke, Eng Guan Chua, Sun Tee Tay, Jamuna Vadivelu

**Affiliations:** ^1^Department of Medical Microbiology, University of Malaya, 50603 Kuala Lumpur, Malaysia; ^2^Faculty of Medicine, University of Malaya, 50603 Kuala Lumpur, Malaysia

## Abstract

Carbapenems are the primary choice of treatment for severe *Pseudomonas aeruginosa* infection. However, the emergence of carbapenem resistance due to the production of metallo-**β**-lactamases (MBLs) is of global concern. In this study, 90 imipenem- (IPM- or IP-) resistant *P. aeruginosa* (IRPA) isolates, including 32 previously tested positive and genotyped for MBL genes by PCR, were subjected to double-disk synergy test (DDST), combined disk test (CDT), and imipenem/imipenem-inhibitor (IP/IPI) *E*-test to evaluate their MBLs detection capability. All three methods were shown to have a sensitivity of 100%. However, DDST was the most specific of the three (96.6%), followed by IP/IPI *E*-test interpreted based on the single criteria of IP/IPI ≥8 as positive (62.1%), and CDT was the least specific (43.1%). Based on the data from this evaluation, we propose that only IRPA with IP MIC >16 *μ*g/mL and IP/IPI ≥8 by IP/IPI *E*-test should be taken as positive for MBL activity. With the new dual interpretation criteria, the MBL IP/IPI *E*-test was shown to achieve 100% sensitivity as well as specificity for the IRPA in this study. Therefore, the IP/IPI *E*-test is a viable alternative phenotypic assay to detect MBL production in IRPA in our population in circumstances where PCR detection is not a feasible option.

## 1. Introduction

Carbapenems, including imipenem (IPM or IP) and meropenem, are the most potent antibacterial agents used for the treatment of infections initiated by multidrug-resistant gram-negative bacilli [1]. However, acquired resistance to carbapenems has been increasingly reported globally, which can be attributed to the evolution of divergent *β*-lactamases in numerous gram-negative bacteria (including *Pseudomonas aeruginosa*). *P. aeruginosa* is an important nosocomial pathogen that is intrinsically resistant to multiple antibiotics. Amongst the various *β*-lactamases that have been identified to date, the genetically mobile metallo-*β*-lactamases (MBLs) are the most versatile ones as they are able to hydrolyse all *β*-lactams except monobactams [2]. Genes encoding for MBL were shown to be carried on large transferable plasmids or were associated with transposons, allowing horizontal transfer of these MBL genes among different bacterial genera and species [3]. To date, five types of acquired MBL genes (IMP, VIM, SPM, GIM, and SIM) have been identified based on their divergent protein molecular structures [4–6]. While IMP and VIM variants have been reported worldwide, members of SPM, GIM, and SIM are restricted to certain geographical regions [7, 8].

Although PCR-based genotyping remains as the golden standard for MBL detection and classification, its use is mainly restricted to research purposes. As genotyping information is necessary, diagnostic centers and laboratories still rely mostly on culture-based phenotypic test as a means for rapid detection of MBL activity. So far, many variations of phenotypic assays for MBLs detection have been reported, and these assays are not standardized. Early detection of MBL-producing organisms is critical as it allows for the prompt use of appropriate antibiotics to effectively control infection. It has been well documented that the activity of MBLs is dependent on zinc or cadmium [4, 9–13]. Several screening methods incorporating the use of metal chelating agents, such as ethylenediaminetetraacetic acid (EDTA) and thiol-based compounds like 2-mercaptopropionic acid (2-MPA), which are capable of blocking MBL activity, have been developed to detect MBL-producing organisms [14–18]. A double-disk synergy test (DDST) using a IPM or ceftazidime (CAZ) disk and a 2-MPA disk, designed by Arakawa et al. [14], was able to indicate the presence of MBLs through the display of an enhanced zone of inhibition around the CAZ disk toward the 2-MPA disk. In addition, a microdilution test [16] and a combined IPM-EDTA disk diffusion method [18] that both utilize EDTA were also developed. The combined IPM-EDTA disk test (CDT) works by comparing the zones of inhibition obtained with IPM disks with and without EDTA [18]. In contrast, the microdilution method compares the minimal inhibitory concentrations (MIC) of IPM with and without EDTA [16]. Both methods were reported to be reliable for the detection of MBLs in carbapenem-resistant *Pseudomonas* and *Acin*et*obacter *strains [16, 18]. Recently a commercial *E*-test strip (AB BioDisk, Solna, Sweden), which offers antimicrobial resistance testing based on the reduction of MICs of IPM in the presence of chelating agents (EDTA), was also developed. The MBL imipenem/imipenem-inhibitor (IP/IPI) *E*-test has been reported to be sensitive for the detection of MBLs in *Acin*et*obacter *species, *P. aeruginosa*, *Serratia* species, *Stenotrophomonas maltophilia*, and *Bacteroides fragilis *[17].

In our recent study we reported that prevalence of IPM-resistant *P. aeruginosa* (IRPA) isolates in Malaysia is high; both IMP and VIM MBLs, but not SIM, GIM, and SPM, were detected in our local isolates [19]. Integron carrying *bla*_VIM_ and *bla*_IMP_ genes in IRPA were found using a PCR-based method [20]. In this study, three phenotypic methods (CDT, DDST, and IP/IPI *E*-test) for MBL detection in IRPA clinical isolates were evaluated in comparison to PCR detection of MBL genes as the gold standard.

## 2. Materials and Methods

### 2.1. Clinical Isolates

A total of 90 IRPA isolated from various clinical specimens of nonrepetitive patients admitted to the University of Malaya Medical Centre (UMMC) were used for this study [6]. These included (i) 5 isolates from Intensive care unit (ICU) and 25 isolates from other wards, collected from October 2005 to March 2006; (ii) 10 isolates from ICU and 50 from other wards, collected from October 2007 to March 2008. These isolates were identified as *P. aeruginosa *by the Medical Microbiology Diagnostic Laboratory using routine biochemical confirmatory tests. MBL gene detection and genotyping was carried out as previously described [6].

### 2.2. Phenotypic Detection of MBL Activity

#### 2.2.1. Combined Disk Test

**Figure 1:**
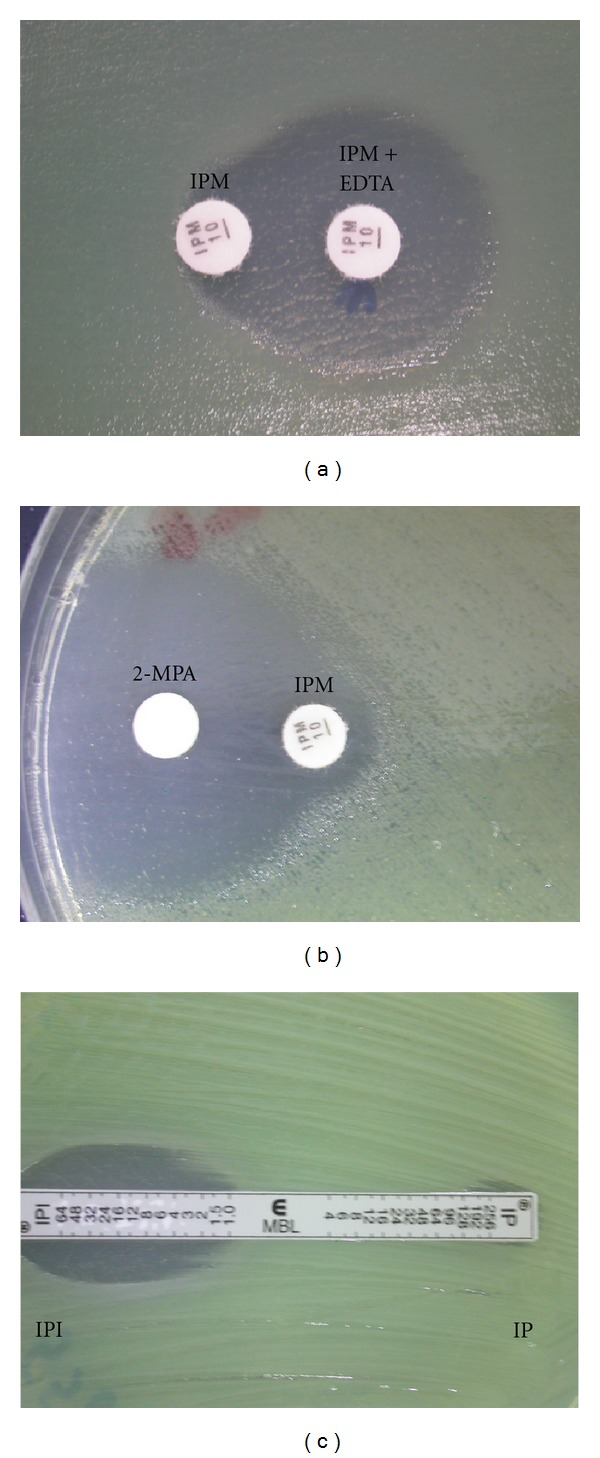
Phenotypic tests to detect MBL production. (a) Combined disk test (CDT) showing enhanced inhibition zone of >7 mm around IPM + EDTA disc indicating MBL positivity. (b) Double-disk synergy test (DDST) with synergistic zone of inhibition surrounding IPM and 2-mercaptopropionic acid (2-MPA) disks indicating MBL activity. (c) MBL IP/IPI *E*-test demonstrating enhanced MIC of imipenem in the presence of EDTA IPI with IP/IPI of ≥8 for MBL activity.

The test was performed as described by Yong et al. [18]. Briefly, an overnight culture of an IRPA clinical isolate was diluted with peptone water (Oxoid, USA) to 10^5^ CFU/mL and spread on Mueller-Hinton (MH) agar (Difco, France) plate using cotton swab. Two IPM disks (Oxoid, UK) were placed on the surface of the agar at a distance of 4-5 cm from each other. 5 *μ*L of 750 *μ*g/mL EDTA solution (Gibco BRL, USA) was then added to one of the IPM disks (Oxoid, UK). The inhibition zones displayed around the IPM (Oxoid, UK) and the IPM-EDTA disks were compared after 14 to 16 hrs of incubation at 37°C (Figure 1(a)). The difference of ≥7 mm between the inhibition zone diameter of the IPM-EDTA disk and that of IPM only disk was considered to be a positive for the presence of MBLs [18]. The procedure was repeated twice to ensure the reproducibility of results.

#### 2.2.2. Double Disk Synergy Test (DDST)

DDST was performed according to Lee et al. [13]. Briefly, an overnight culture of an IRPA clinical isolate was diluted with peptone water (Oxoid, USA) to 10^5^ CFU/mL and spread on MH agar (Difco, France) plate using cotton swab. Two IPM disks (Oxoid, UK) were placed on the surface of the agar 4-5 cm (center to center) apart. A blank filter disk (Oxoid, UK) was subsequently placed near one of the IPM disks (Oxoid, UK) at a distance from 1.0 to 1.5 cm, and 3 *μ*L of 2-MPA (Sigma, USA) was applied onto the blank disk. The plate was incubated overnight. The presence of a synergistic inhibitory zone was regarded as MBL positive (Figure 1(b)).

#### 2.2.3. MBL IP/IPI *E*-Test

An overnight culture of an IRPA clinical isolate was diluted in peptone water to a turbidity of a 0.5 McFarland standard. A cotton swab was then used to transfer the inoculum onto a MH agar plate. Once dried, an *E*-test MBL strip (AB BioDisk, Solna, Sweden) was applied onto the plate which was then incubated at 37°C for 16 to 18 hrs to detect the presence of metalloenzymes. Interpretation of results was carried out according to the manufacturer's instructions. A reduction in MIC in the presence of EDTA of greater than or equal to eight-fold (IP/IPI ≥ 8) is interpreted as indicating MBL activity (Figure 1(c)).

### 2.3. Statistical Analysis

Statistical analysis was carried out using two-tailed Student's *t*-test with *P* < 0.001 considered significant. Sensitivity was determined as number of true positives/(number of true positives + number of false negatives). Specificity was calculated as number of true negatives/(number of true negatives + number of false positives). Cutoff values were determined using the SPSS software (IBM, USA) graphically displayed as a receiver-operating characteristic (ROC) curve. 

## 3. Results and Discussion 

Among 90 IRPA clinical isolates used in this study, MBL genes (*bla*_IMP-4,  IMP-7_, *bla*_VIM-2, VIM-11_) were detected in 32 isolates by PCR. In addition, these 32 isolates were also found to be resistant to at least six antibiotics and, hence, regarded as multidrug-resistant isolates. 

In the evaluation of three selected MBL phenotypic assays (CDT, DDST, and IP/IPI *E*-test) (Table 1), all three methods were shown to have a sensitivity of 100%. However, specificity of phenotypic assays differs. DDST was the most specific of the three (96.6%), followed by IP/IPI *E*-test with the single criteria of IP/IPI ≥ 8 as positive (62.1%). CDT was the least specific (43.1%). In all three phenotypic assays, false-positive MBL producers were detected. These false-positive cases might actually be producing an unknown and weaker *β*-lactamases, which is worth further investigation. Here, we demonstrated that DDST was more specific in detecting MBLs in comparison to the CDT as also previously described by Lee et al. [13] and Pitout et al. [12]. On the other hand, this is unlike Qu et al. [21] who demonstrated that the CDT is the best method for screening for MBL production in *P. aeruginosa* from China. This discrepancy in findings may be due to differences in population structure of MBL genes between different geographical areas (predominantly VIM-2 and IMP-9 in China versus predominantly VIM-2 and IMP-7 in Malaysia). Furthermore, there is no significant difference observed in zone diameter increases for VIM-2-producing *P. aeruginosa* compared to those for IMP-7-producing isolates. 

**Figure 2:**
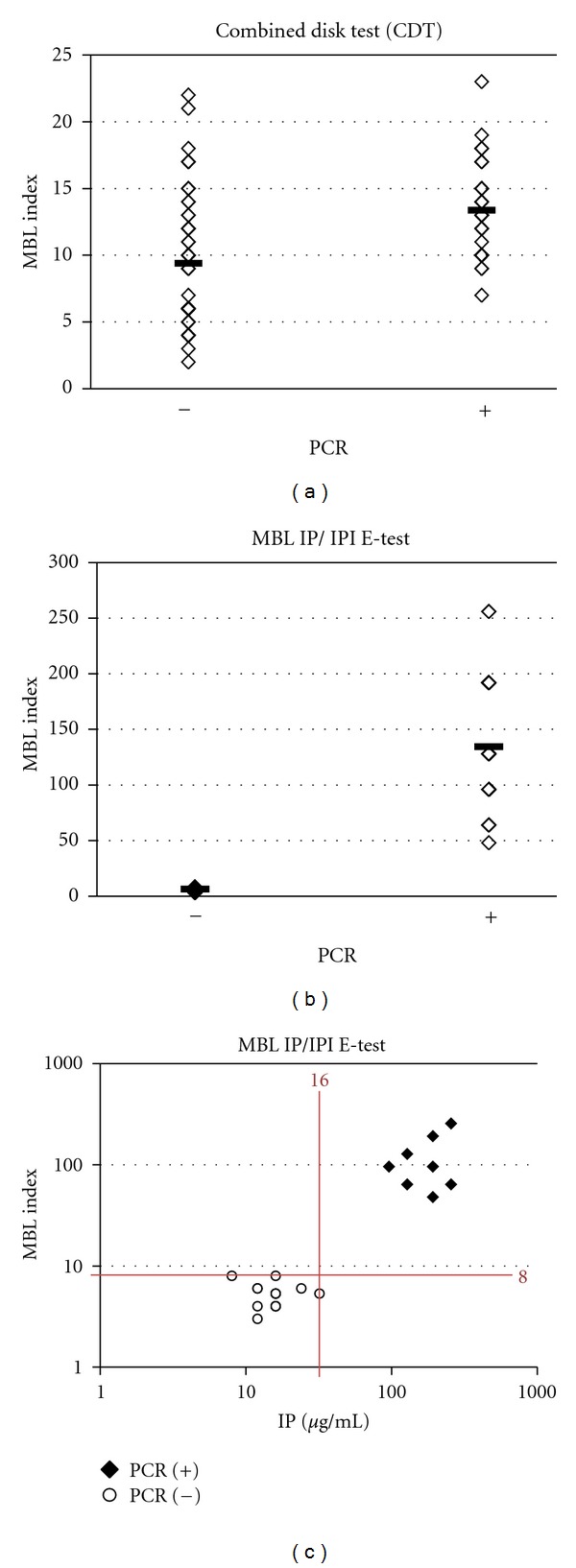
Correlation analysis of combined disk test (CDT), MBL IP/IPI *E*-test, and PCR detection of MBL genes. (a) MBL index by CDT was 9.5 and 13.4 for IRPA isolates that were negative and positive for MBL genes, respectively (*P* < 0.001). (b) MBL index by IP/IPI *E*-test was 6.4 and 134.5 for IRPA isolates that were negative and positive for MBL genes, respectively (*P* < 0.001). Statistical analysis was carried out using two-tailed Student's *t*-test with *P* < 0.001 considered significant. (c) Strong correlation between IP MIC and MBL index with PCR detection of MBL genes. Together, IP > 16 *μ*g/mL and MBL index ≥ 8 were able to distinguish IRPA with MBL genes from those without. The proposed cutoff values were determined from a receiver-operating characteristic (ROC) curve to obtain a 100% sensitivity and specificity for this data set.

Unlike DDST, which is qualitative, CDT and IP/IPI *E*-test are both semiquantitative in nature and enabled the calculation of an MBL index. The MBL index of CDT and IP/IPI *E*-test is an estimate of the relative level of MBL activity and is comparable within the method. However, since there are considerable differences in methodology between CDT and IP/IPI *E*-test, the MBL indexes obtained by these two methods are incomparable. The main advantage of the IP/IPI *E*-test has been the only method among the three studied that allows the MIC to be determined. Although significant differences (*P* < 0.001) exist between the MBL indexes of CDT (Figure 2(a)) and IP/IPI *E*-test (Figure 2(b)) as compared direct detection of MBL genes by PCR, distinct subsets were obvious only with IP/IPI *E*-test between IRPA with and without MBL genes (Figure 2(c)). 

The high false-positive reporting rate attributed to CDT is not surprising as the isolates tested in this study were IPM resistant, and, therefore, the inhibition zone of ≥7 mm in zone diameter in the presence of EDTA may not be considered as a definitive clear cutoff criterion to differentiate between MBL-producing and non-MBL-producing IPRA isolates. In view of that, Yong et al. [18] reported that the best separation between MBL-positive and MBL-negative isolates was obtained using a breakpoint of ≥8 mm in the presence of 750 *μ*g of EDTA instead. It is also important to note that EDTA has membrane-permeabilising properties and could exert a deleterious effect on *P. aeruginosa*; thus, the extended zone size difference between the IPM and IPM-EDTA disks in the CDT may be due to the susceptibility of the organism to EDTA rather than its metal-chelating effect that inactivates any MBL, thus resulting in false-positive detection [22]. 

In the present study, in accordance with our previous PCR findings, the MBL IP/IPI *E*-test was demonstrated to exhibit 100% accuracy in the detection of MBL production. Our results were similar to the findings of Walsh et al. [17] and also in another study on *Acin*et*obacter baumannii* by Segal and Elisha [23]. Since true MBL-producing IRPA tends to be more highly resistant to IPM (higher MIC) than non-MBL-producing IRPA, our results suggest that those isolates with IP MIC < 16 *μ*g/mL be excluded from the determination of MBL status by the IP/IPI *E*-test. In other words, we suggest that only IP MIC > 16 *μ*g/mL (proposed new criteria) together with IP/IPI ≥ 8 (criteria by manufacturer) by *E*-test should be taken as MBL activity-positive for IRPA isolated from Malaysian patients. With the new dual criteria, the MBL IP/IPI *E*-test was able to achieve 100% sensitivity and 100% specificity. A larger-scale study involving more IRPA strains with larger geographical scope will be needed to verify the validity of the new proposed criteria for interpreting the MBL IP/IPI *E*-test. 

Despite the accuracy of MBL IP/IPI *E*-test detection, it is costly compared to the antibiotic disks to be used by health care institutions or clinical laboratories for routine MBL screening procedure. DDST, which exhibited up to 96.6% specificity, is perhaps a more suitable routine screening procedure to be considered for early detection of MBL-producing bacteria. However, in order to minimize false positivity, isolates positive by DDST can be further confirmed by MBL IP/IPI *E*-test. 

In conclusion, MBL detection remains a controversial issue, and clinical laboratories are in need of a simple and direct method to recognize such resistance in gram-negative bacteria to improve disease management. Furthermore, in recognition that MBL genotypes are not homogenous in geographical distribution, a generalized criteria for interpretation of MBL phenotypic assays may not be possible. Thus, it is recommended that the phenotypic assays should be assessed and adopted based on the local situation. 

## Figures and Tables

**Table 1:** Detection of MBL activity and MBL genes of IRPA clinical isolates from Malaysia.

Bacterial strain	CDT				DDST	IP/IPI *E*-test				PCR
	IMP (mm)	IMP + EDTA (mm)	MBL index		MBL index	IP (*μ*g/mL)	IPI (*μ*g/mL)	MBL index		
Ps1	10	22	12	+	+	256	1	256	+	IMP-7
Ps2	12	30	18	+	+	256	4	64	+	IMP-4
Ps3	10	24	14	+	+	256	1	256	+	IMP-4
Ps4	10	22	12	+	+	192	<1	>192	+	VIM-2
Ps5	10	20	10	+	+	192	1	192	+	VIM-2
Ps6	10	24	14	+	+	192	<1	>192	+	VIM-2
Ps7	10	25	15	+	+	192	<1	>192	+	VIM-2
Ps8	10	20	10	+	+	192	<1	>192	+	VIM-2
Ps9	11	21	10	+	+	192	<1	>192	+	VIM-2
Ps10	11	26	15	+	+	192	1	192	+	VIM-2
Ps11	10	24	14	+	+	192	<1	>192	+	VIM-2
Ps12	12	21	9	+	+	192	2	96	+	VIM-2
Ps13	12	22	10	+	+	192	2	96	+	VIM-2
Ps14	10	22	12	+	+	192	<1	>192	+	IMP-7
Ps15	12	30	18	+	+	192	4	48	+	IMP-7
Ps16	10	23	13	+	+	192	2	96	+	IMP-7
Ps17	10	23	13	+	+	192	2	96	+	IMP-7
Ps18	10	27	17	+	+	192	2	96	+	IMP-7
Ps19	10	17	7	+	+	192	2	96	+	IMP-7
Ps20	10	23	13	+	+	128	2	64	+	VIM-2
Ps21	10	25	15	+	+	128	<1	>128	+	VIM-2
Ps22	10	29	19	+	+	128	<1	>128	+	VIM-2
Ps23	10	27	17	+	+	128	1	128	+	VIM-11
Ps24	10	20	10	+	+	128	<1	>128	+	VIM-2
Ps25	10	33	23	+	+	128	2	64	+	VIM-2
Ps26	10	27	17	+	+	128	2	64	+	VIM-2
Ps27	11	21	10	+	+	128	2	64	+	VIM-2
Ps28	12	30	18	+	+	128	1	128	+	IMP-7
Ps29	12	21	9	+	+	128	1	128	+	IMP-7
Ps30	11	21	10	+	+	128	1	128	+	IMP-7
Ps31	10	23	13	+	+	128	1	128	+	IMP-7
Ps32	11	22	11	+	+	96	<1	>96	+	IMP-7
Ps33	10	22	12	+	−	32	6	5	−	−
Ps34	12	18	6	−	−	24	4	6	−	−
Ps35	10	27	17	+	−	24	4	6	−	−
Ps36	10	25	15	+	−	24	4	6	−	−
Ps37	12	23	11	+	−	24	4	6	−	−
Ps38	13	25	12	+	−	24	4	6	−	−
Ps39	10	19	9	+	−	24	4	6	−	−
Ps40	10	18	5	+	−	24	4	6	−	−
Ps41	10	25	15	+	−	24	4	6	−	−
Ps42	12	18	6	−	−	24	4	6	−	−
Ps43	10	20	10	+	−	24	4	6	−	−
Ps44	10	25	15	+	+	24	4	6	−	−
Ps45	9	30	21	+	−	24	4	6	−	−
Ps46	10	24	14	+	−	24	4	6	−	−
Ps47	10	16	6	−	−	24	4	6	−	−
Ps48	12	24	12	+	−	24	4	6	−	−
Ps49	13	20	7	+	−	24	4	6	−	−
Ps50	12	18	6	−	−	16	2	8	+	−
Ps51	10	20	10	+	+	16	2	8	+	−
Ps52	12	18	6	−	−	16	3	5	−	−
Ps53	11	21	10	+	−	16	3	5	−	−
Ps54	10	23	13	+	−	16	3	5	−	−
Ps55	13	25	12	+	−	16	4	4	−	−
Ps56	11	24	13	+	−	16	3	5	−	−
Ps57	11	21	10	+	−	16	4	4	−	−
Ps58	10	27	17	+	−	16	4	4	−	−
Ps59	12	23	11	+	−	16	3	5	−	−
Ps60	10	16	6	−	−	16	3	5	−	−
Ps61	10	22	12	+	−	16	4	4	−	−
Ps62	10	32	22	+	−	16	3	5	−	−
Ps63	10	22	12	+	−	16	4	4	−	−
Ps64	10	20	10	+	−	16	4	4	−	−
Ps65	11	21	10	+	−	12	2	6	−	−
Ps66	14	20	6	−	−	12	2	6	−	−
Ps67	13	18	5	−	−	12	2	6	−	−
Ps68	10	13	3	−	−	12	3	4	−	−
Ps69	12	14	2	−	−	12	4	3	−	−
Ps70	10	27	17	+	−	12	2	6	−	−
Ps71	10	20	10	+	−	8	<1	>8	+	−
Ps72	12	21	9	+	−	8	<1	>8	+	−
Ps73	12	21	9	+	−	8	<1	>8	+	−
Ps74	10	24	14	+	−	8	<1	>8	+	−
Ps75	10	14	4	−	−	8	<1	>8	+	−
Ps76	12	30	18	+	−	8	<1	>8	+	−
Ps77	12	18	6	−	−	8	<1	>8	+	−
Ps78	12	18	6	−	−	8	<1	>8	+	−
Ps79	10	16	6	−	−	8	1	8	+	−
Ps80	11	17	6	−	−	8	1	8	+	−
Ps81	12	18	6	−	−	8	<1	>8	+	−
Ps82	12	18	6	−	−	8	<1	>8	+	−
Ps83	14	18	4	−	−	8	<1	>8	+	−
Ps84	13	17	4	−	−	8	<1	>8	+	−
Ps85	13	19	6	−	−	8	<1	>8	+	−
Ps86	12	18	6	−	−	8	1	8	+	−
Ps87	12	18	6	−	−	8	1	8	+	−
Ps88	10	14	4	−	−	8	<1	>8	+	−
Ps89	11	15	4	−	−	8	<1	>8	+	−
Ps90	12	17	5	−	−	8	<1	>8	+	−
