# Corrigendum to “Phenotypic Detection of Metallo-*β*-Lactamase in Imipenem-Resistant *Pseudomonas aeruginosa*”

**DOI:** 10.1155/2016/9562039

**Published:** 2016-05-24

**Authors:** Yalda Khosravi, Mun Fai Loke, Eng Guan Chua, Sun Tee Tay, Jamuna Vadivelu

**Affiliations:** ^1^Department of Medical Microbiology, University of Malaya, 50603 Kuala Lumpur, Malaysia; ^2^Faculty of Medicine, University of Malaya, 50603 Kuala Lumpur, Malaysia

The Acknowledgments section of the article titled “Phenotypic Detection of Metallo-*β*-Lactamase in Imipenem-Resistant* Pseudomonas aeruginosa*” [1] has been revised as follows: This paper is supported by University of Malaya-Ministry of Education (UM-MoE) High Impact Research (HIR) Grant UM.C/625/1/HIR/MoE/CHAN-02 (Account no. H-50001-A000013) and University of Malaya PPP Grant (Vote PS296/2007B). The authors would like to thank Professor Emeritus Yunsop Chong and Professor Kyungwon Lee from the Department of Clinical Pathology and Research Institute of Bacterial Resistance, Yonsei University College of Medicine, Seoul, Republic of Korea, for kindly providing the IMP-1, SIM-1, and VIM-2 *β*-lactamase producing* Acinetobacter* spp. and* P. aeruginosa* strains for use as positive controls.
